# The Impact of Clinical Prognosis of Viral Hepatitis in Head and Neck Cancer Patients Receiving Concurrent Chemoradiotherapy

**DOI:** 10.3390/biomedicines11112946

**Published:** 2023-11-01

**Authors:** Yu-Ming Wang, Sheng-Dean Luo, Ching-Nung Wu, Shao-Chun Wu, Wei-Chih Chen, Yao-Hsu Yang, Tai-Jan Chiu

**Affiliations:** 1 Department of Radiation Oncology & Proton and Radiation Therapy Center, Kaohsiung Chang Gung Memorial Hospital, Chang Gung University College of Medicine, Kaohsiung 833, Taiwan; scorpion@cgmh.org.tw; 2 School of Traditional Chinese Medicine, College of Medicine, Chang Gung University, Taoyuan 333, Taiwan; rsd0323@cgmh.org.tw (S.-D.L.); r95841012@cgmh.org.tw (Y.-H.Y.); 3 Department of Otolaryngology, Kaohsiung Chang Gung Memorial Hospital, Chang Gung University College of Medicine, Kaohsiung 833, Taiwan; taytay@cgmh.org.tw (C.-N.W.); jarva@cgmh.org.tw (W.-C.C.); 4 Graduate Institute of Clinical Medical Sciences, College of Medicine, Chang Gung University, No. 259, Wenhua 1st Rd., Guishan District, Taoyuan 333, Taiwan; sean1207@cgmh.org.tw; 5 Department of Anesthesiology, Kaohsiung Chang Gung Memorial Hospital, Chang Gung University College of Medicine, Kaohsiung 833, Taiwan; 6 Department of Traditional Chinese Medicine, Chang Gung Memorial Hospital, Chiayi 613, Taiwan; 7 Health Information and Epidemiology Laboratory of Chang Gung Memorial Hospital, Chiayi 613, Taiwan; 8 Division of Hematology-Oncology, Department of Internal Medicine, Kaohsiung Chang Gung Memorial Hospital, Chang Gung University College of Medicine, Kaohsiung 833, Taiwan

**Keywords:** head and neck cancer, viral hepatitis, chemoradiation, antiviral therapy, prognosis

## Abstract

This study evaluated the clinical characteristics of head and neck cancer (HNC) patients with hepatitis B (HBV) or hepatitis C (HCV) who underwent concurrent chemoradiotherapy (CCRT) and examined the prognostic impact of antiviral therapies. In a 19-year retrospective analysis of 8224 HNC patients treated with CCRT, 29.8% (2452) were diagnosed with HBV or HCV, of whom 714 received antiviral therapy. For non-metastatic HNC patients on CCRT, factors such as gender, Charlson Comorbidity Index (CCI), liver cirrhosis markers (Fibrosis-4, APRI), and initial tumor stage were significant determinants of their overall survival. However, the presence of HBV or HCV and the administration of antiviral treatments did not yield distinct survival outcomes. In summary, antiviral therapy for HBV or HCV did not affect the 5-year survival rates of non-metastatic HNC patients undergoing CCRT, while gender, tumor stage, CCI, and liver cirrhosis were notable prognostic indicators.

## 1. Introduction

Head and neck cancers (HNC) include malignancies in the oral cavity, oropharynx, hypopharynx, larynx, nose, and sinus. The most common cell type of HNC is squamous cell carcinoma. Given the predicted rising incidence, HNC remains a significant public health threat with high morbidities rates and the most cancer-related death worldwide. Clinically, only 30–40% of HNC patients initially presented with early tumors that are often cured with complete tumor resection or definitive chemoradiotherapy for functional preservation [1]. For most advanced HNC patients, concurrent chemoradiotherapy (CCRT) is better at improving the local control rate, organ preservation, and lowering the distant metastases rate than radiotherapy on its own [2,3,4]. Platinum-based CCRT is the principal treatment of choice, despite the availability of novel effective agents. However, the acute and late adverse effects of CCRT impede HNC patients’ overall survival due to competing morbidities.

Both hepatitis B (HBV) and hepatitis C virus (HCV) infections are major public health problems affecting millions of people worldwide. Without treatment, many patients with chronic HBV and HCV infections will develop liver-related morbidities, such as cirrhosis, hepatocellular carcinoma, and die within 20–30 years [5,6]. In oncology, chemotherapy might induce immunosuppression and reactivate a quiescent hepatitis virus. Previous studies have reported reactivation rates of 30–80% in HBV and 10–36% in HCV [7,8]. The American Association of Study in Liver Diseases (AASLD) and the European Association for the Study of Liver Disease (EASL) have recommended HBV testing and administering antiviral prophylaxis before starting chemotherapy for HBsAg-positive patients with solid tumors [9,10,11]. In contrast to hepatitis B, there is no guideline for cancer patients with HCV infection receiving chemotherapy.

HBV and HCV infections are endemic in Taiwan [12,13]. According to the Taiwan Cancer Registry Annual Report in 2018, HNC had the fourth-highest incidence rate of all cancer types. More than 50% of HNC patients received radiotherapy and chemotherapy—even post complete tumor resection. In the literature review, studies of the impact of an HBV or HCV infection on HNC patients receiving CCRT are limited. Therefore, we aimed to investigate the prognosis and efficacy of anti-HBV or HCV treatments in HNC patients receiving CCRT.

## 2. Materials and Methods

### 2.1. Patient Recruitment

This retrospective study was reviewed and approved by the Institutional Review Board (IRB) of the Kaohsiung and Chiayi branches of Chang Gung Memorial Hospital. According to these protocols, informed consent was waived due to the study design and IRB regulations. All methods were performed following the relevant guidelines and regulations. The IRB approval protocol number is 202001132B0C601.

### 2.2. Study Design and Subjects

A total of 35,910 patients were diagnosed with HNC in four Chang-Gung Memorial Hospitals, Taiwan, between January 2001 and December 2019. Diagnoses of HNC were based on the typical findings detected via computed tomography (CT) or magnetic resonance imaging (MRI) and pathological reports. Patients should have received abdominal sonography and bone scans or positron emission tomography for complete tumor staging. The inclusion criteria were as follows: (i) primary HNC without metastasis; (ii) being older than 18-years-old; and (iii) receiving CCRT. The exclusion criteria included: (i) missing data on stages of cancer (pathological AJCC or clinical TN staging system); (ii) only undergoing chemotherapy or only radiotherapy; (iii) undergoing a radiotherapy with non-curative intent; and (iv) patients who died within one year after initial diagnosis. The criterion for recruiting patients in this study was those who had HBV or HCV, but not concomitant HBV and HCV. In total, 8224 HNC patients who underwent CCRT were analyzed in our study. We defined an HBV or HCV infection as the presence of any disease codes from 2001 to 2019 (HBV: B18.0, B18.1, B18.10, B18.18, or Z22.5; HCV: B18.2), and the diagnostic accuracy was validated.

### 2.3. Treatment

Radiotherapy: For the patients treated with conventional two-dimensional radiotherapy, the radiation doses applied were 70–76 Gy for the primary tumor, 60–70 Gy for neck lymph nodes showing malignancy, and 50 Gy for the non-affected lymphatics of the neck. For the patients treated with intensity-modulated radiation therapy (IMRT), the dose guidelines were 69–72 Gy for the primary tumor and gross lymph nodes, 60–65 Gy for the high-risk prophylaxis area, and 50–56 Gy for the low-risk prophylaxis area. The treatment was consistently delivered in daily fractions over a five-day week for all patients.

Chemotherapy: Cisplatin 30 to 40 mg/m^2^ weekly or carboplatin two cycles of carboplatin (area of the curve (AUC) of 5).

Antiviral treatment: Patients with positive HBsAg results for at least six months received prophylactic antiviral therapy one week before the initiation of chemotherapy. HBsAg(+) patients were administered antiviral drugs: 0.5 mg of an Entecavir (ETV) tablet once daily, 100 mg of an lamivudine tablet once daily, or 10 mg of an adefovir tablet once daily according to a hepatologists’ prescription. According to the reimbursement policy of Taiwan’s National Health Insurance (NHI), anti-hepatitis B treatment can be continued for at least six months after the completion of chemotherapy. From 2004 to 2011, the IFN-based therapy and pegylated interferon plus ribavirin (PegIFN/RBV) (180 microg/wk, for 24 or 48 weeks plus a standard weight-based dose (1000 or 1200 mg/d) of ribavirin) was the standard treatment for HCV infection. Direct-acting Antiviral Agents (DAAs) in hepatitis C therapy have been reimbursed by Taiwan’s NHI since 2017 for HCV viremic patients regardless of the patient’s liver fibrosis status [14].

For the enrolled patients, comorbidity was also defined using ICD-10 codes and summarized using the Charlson Comorbidity Index (CCI) score [15,16]. We use the fibrosis-4 (FIB-4) index and aspartate-aminotransferase-to-platelet ratio index (APRI) score as the baseline to assess the degree of liver disease. Scores of APRI > 2 and FIB-4 > 3.25 were defined as advanced fibrosis [17].

We traced the electronic records of HNC patients who received CCRT within a 10-year follow-up period. Not all the patients with HBV infection had HBV DNA-level data at the baseline. HBV or HCV reactivation could not be determined and was not included as an outcome measure. Therefore, the incidence measure was the severe acute exacerbation of an HBV or HCV infection. Severe acute exacerbation of an HBV or HCV infection was defined as (1) when the serum alanine aminotransferase (ALT) level increased beyond 10 times the normal upper limit (ALT level ≥ 400 IU/L) during chemotherapy or 6 months following chemotherapy; (2) the presence of a serum hepatitis B surface antigen or anti-HCV antibody at acute exacerbation; and (3) the exclusion of liver damage causes, such as a superinfection or coinfection with hepatitis A and D viruses, alcoholic liver disease, autoimmune hepatitis, drug-induced hepatitis, or major systemic conditions such as shock, hypoxia, or hemolytic anemia. Any suspected instances of severe acute exacerbation were thoroughly reviewed by a dedicated hepatic injury panel in this study. This review took into account the patient’s clinical progress, serological markers, and HBV DNA levels at the time of the acute event.

### 2.4. Statistical Analysis

Categorical variables such as the patients’ age, gender, comorbidities, FIB-4 index, and AJCC cancer stages were evaluated using either a two-sided Fisher’s exact test or Pearson’s chi-squared test. Continuous data, depending on their distribution, were analyzed with either Student’s two-tailed *t*-tests (for normally distributed data) or Mann–Whitney U tests (for non-normally distributed data). The Kaplan–Meier method assessed the impact of antiviral treatment on primary outcomes (OS), accounting for confounders such as gender, age, and AJCC cancer stages. Univariate analysis and the Cox proportional-hazards model identified the covariates influencing the survival of HNC patients. All analyses were conducted using IBM SPSS Statistics for Windows, Version 22.0. Armonk, NY, USA.

## 3. Results

### 3.1. Demographic and Clinical Characteristics of Patients Diagnosed with HNC

**Figure 1:**
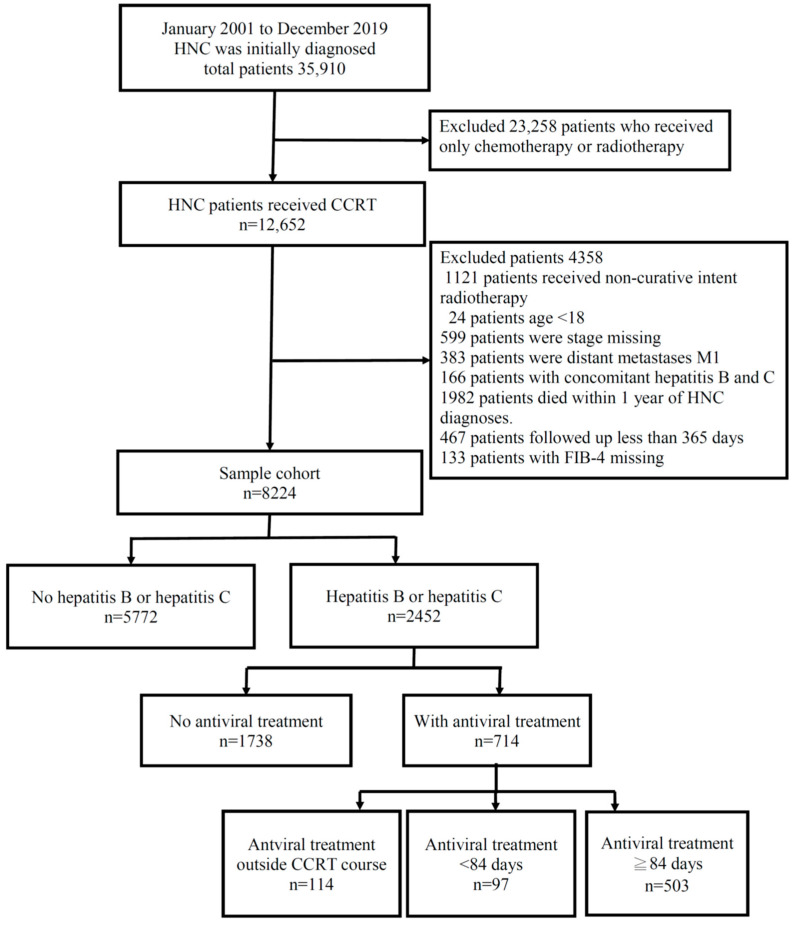
Flow chart showing the inclusion and exclusion criteria of the HNC patients receiving CCRT recruited in this study. A total of 8224 patients diagnosed with HNC were enrolled in this study and 2452 patients were enrolled with hepatitis B or hepatitis C infection. Among these patients, 1852 patients did not receive an antiviral treatment in the CCRT course, and 600 patients did receive an antiviral treatment in the CCRT course.

Figure 1 presents a flow chart depicting HNC patients who underwent CCRT at Chang Gung Memorial Hospital in Taiwan from January 2001 to December 2019. Of the 8224 HNC patients treated with CCRT, 2452 were diagnosed with either hepatitis B or hepatitis C infection. Notably, 600 of these patients received antiviral treatment during their CCRT course. The baseline characteristics of this study are shown in Table 1.

In this study, the median age was 52 years old, and 88.07% (7243) of the HNC patients were male. The primary sites include the oral cavity (2599, 31.6%), oropharynx (1321, 16.1%), hypopharynx (1111, 13.5%), larynx (498, 6.1%), and others (32.8%). A total of 2452 patients had viral hepatitis, and 600 received an antiviral treatment during the CCRT. In all, 1415 patients had stage I or II AJCC. A total of 1631 patients had stage III AJCC. A total of 3825 patients had stage IVA, and 1353 patients had stage IVB. There were 8143 patients with a CCI score of 0–5 and 83 patients with a CCI score ≥ of 6. A total of 8124 patients had an APRI score < 2, 100 patients had an APRI score ≥ 2.7869 patients had an FIB-4 score < 3.25, and 355 patients had an FIB-4 score ≥ 3.25.

### 3.2. Univariate and Multivariate Analyses of Predictive Variables for HNC Survival

**Figure 2:**
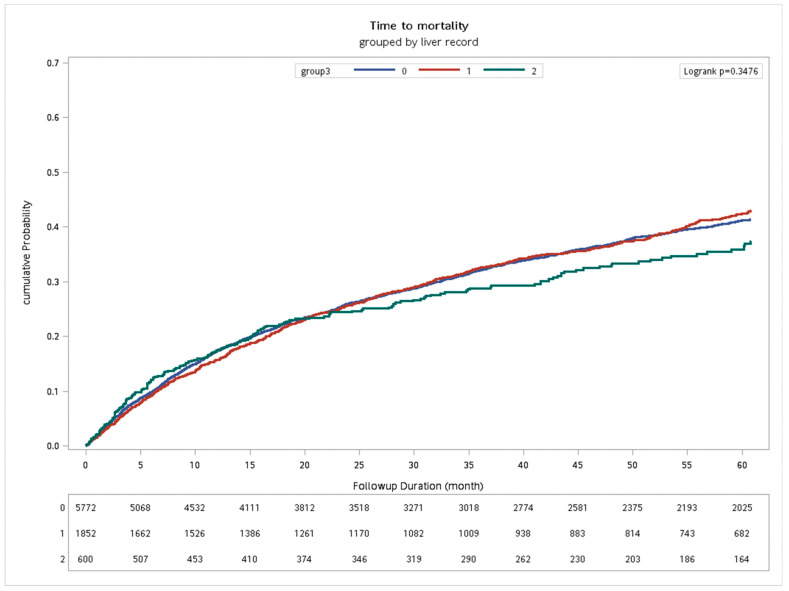
The Kaplan–Meier curve of time to mortality of three groups of HNC patients receiving CCRT. Group 1: no HBV or HCV infection (*n* = 5772); Group 2: HBV or HCV infection without antiviral treatment in CCRT course (*n* = 1852); Group 3: HBV or HCV infection with antiviral treatment in CCRT course (*n* = 600).

In model A, univariate Cox regression revealed significant overall survival (OS) prognostic factors for the HNC patients who were receiving CCRT, including age (≥52 vs. <52 y/o, HR = 1.19, *p* < 0.0001), gender (female vs. male, HR = 0.46, *p* < *0*.0001), primary tumor site, T stage, N stage, AJCC tumor stage, CCI (≥6 vs. 0–5, HR = 2.19, *p* < 0.0001), APRI (≥2 vs. <2, HR = 1.68, *p* = 0.0002), and FIB-4 (≥3.14 vs. <3.14, HR = 2.04, *p* < 0.0001). After adjusting for confounders, Cox regression highlighted gender, primary site, T stage, N stage, AJCC tumor stage, CCI, APRI, and FIB-4 as independent prognostic factors for OS. Notably, no survival difference was observed among three HNC groups on CCRT: (1) those without hepatitis B or C, (2) those who received an antiviral treatment for hepatitis B or C, and (3) those who did not undergo such treatment (Table 2, Figure 2).

**Figure 3:**
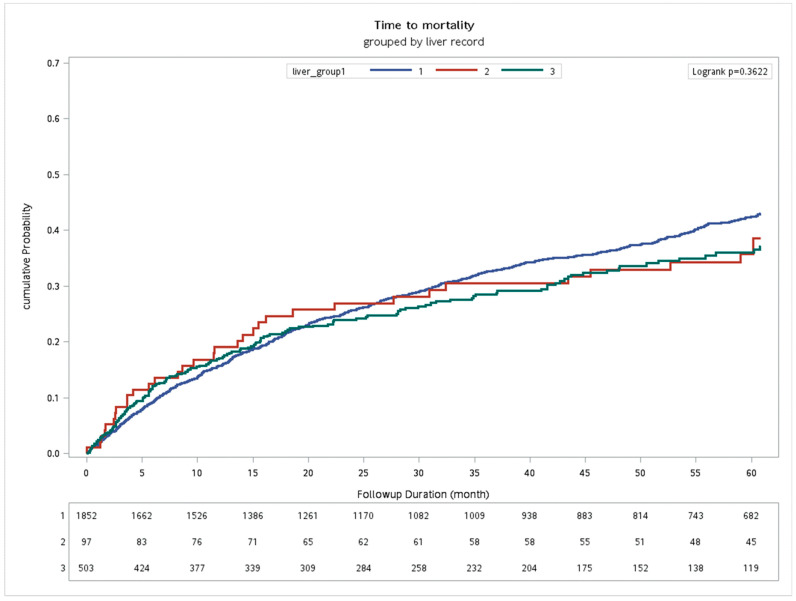
The Kaplan–Meier curve of time vs. mortality of three groups of HNC patients with HBV or HCV infection receiving CCRT (*n* = 2452). Group 1: HBV or HCV infection without antiviral treatment in CCRT course (*n* = 1852); Group 2: HBV or HCV infection with antiviral therapy < 84 days in CCRT course (*n* = 97); Group 3: HBV or HCV with antiviral therapy ≥ 84 days in CCRT course (*n* = 503).

To evaluate the outcome of antiviral treatment in HNC patients with hepatitis B or C, we allocated 2452 HNC patients with hepatitis B or C who received CCRT into the 3 groups: (1) those not given the antiviral treatment, (2) those given the antiviral treatment <84 days, and (3) those given the antiviral treatment ≥84 days (model B). The univariate Cox regression of the model B revealed that the gender, primary tumor site (hypopharynx and others), T stage, N stage, AJCC tumor stage of cancer (stage IVA and stage IVB), CCI score, APRI score, and FIB-4 were prognostic factors in HNC patients with hepatitis receiving CCRT (Table 3). In adjusted multivariate Cox regression analysis, significant prognostic factors on OS for HNC patients with hepatitis receiving CCRT were gender (female vs. male, HR = 0.67, *p* = 0.0159), primary tumor site (hypopharynx vs. oral, HR = 1.57, *p* < 0.0001; others vs. oral, HR = 0.60, *p* < 0.0001), T stage (stage 3 vs. stage 1, HR = 1.60, *p* = 0.0007; stage 4 vs. stage 1, HR = 1.82, *p* < 0.0001), N stage (stage 2 vs. stage 0, HR = 1.31, *p* = 0.0025; stage 3 vs. stage 0, HR = 2.30, *p* < 0.0001), AJCC stage (stage IVA vs. stage I/II, HR = 1.79, *p* < 0.0001; stage IVB vs. stage I/II, HR = 2.73, *p* < 0.0001), CCI (≥6 vs. 0–5, HR = 1.83, *p* = 0.0028), APRI (≥2 vs. <2, HR = 1.65, *p* = 0.0013), and FIB-4 (≥3.14 vs. <3.14, HR = 1.84, *p* < 0.0001). In model B, there was no survival difference between the HNC patients with hepatitis receiving the antiviral infection treatment or not during the CCRT period (Table 3 and Figure 3).

From the above studies, there was no survival difference in these three groups of HNC patients receiving CCRT: (1) no hepatitis B or C, (2) hepatitis B or C without antiviral treatment during CCRT, and (3) hepatitis B or C with antiviral treatment in the CCRT course. We retrospectively reviewed the electronic medical records of 3014 HNC patients with hepatitis B or C to evaluate the incidence of acute hepatitis during CCRT. These patients included an overall survival time of less than one year. Only 44 HNC patients (1.45%) with hepatitis B or C experienced severe acute exacerbation during CCRT, and 14 died between January 2001 and December 2019. The most common cause of death was liver metastases, and none of them died of fatal HBV or HCV reactivation.

## 4. Discussion

A previous study showed that cancer patients with chronic hepatitis B infection or hepatitis C infection who received chemotherapy might have a higher mortality rate due to HBV or HCV reactivation [18]. The HBV reactivation rates were from 14% to 72% in the HBV carriers who received different chemotherapy agents and the oncologic patients with different HBV serologic statuses [18]. A total of 23% of the HCV carriers also might have undergone HCV reactivation while receiving cancer-related treatments, although these patients were usually asymptomatic [19]. The cancer patients with reactivated HBV and HCV experienced liver cirrhosis, hepatic encephalopathy, and even fulminant liver failure and death. The American Society of Clinical Oncology commented that cancer patients should examine their HBV serologic status before they intend to receive chemotherapy to prevent HBV reactivation [20]. However, some questions about HBV and HCV infections need elucidation before chemotherapy is given to cancer patients. From the literature review, it seems there is insufficient evidence to support the HCV or HBV screening and antiviral treatment of HNC patients before they receive chemotherapy.

In some retrospective studies, HNC patients had higher HBV or HCV infection rates [21,22]. In Asia, especially South Asia, HNC has caused increased incidence and mortality rates, and, most significantly, a national burden [23]. However, from the literature review, only a few small studies evaluated the prognosis of hepatitis B or C infection in HNC patients receiving CCRT. In Taiwan, national health insurance reimbursed cancer patients for anti-HBV treatment before and during receiving chemotherapy since 2009, including HNC patients. In our research, 8143 HNC patients received CCRT at Chang-Gung Memorial hospitals in Keelung, Taoyuan, Chiayi, and Kaohsiung from 2001 to 2019. Hepatitis B or C infection did not impact the HNC patients’ overall survival during CCRT. In model A, the OSs of the HNC patients in the groups with hepatitis who did or did not receive the antiviral treatment were similar to a group of patients without HBV or HCV infection. In addition, the antiviral therapy did not affect the HNC patients’ outcomes in model B. The duration of antiviral treatments (<84 days and ≥84 days) did not improve the OS of the HNC patients without antiviral therapy for their HBV or HCV during CCRT. The effect of the antiviral treatment of HBV or HCV carriers in HNC patients to receive CCRT is different from that of other malignancies, such as lymphoma or breast cancer, after chemotherapy, or target therapy. The incidence of severe acute exacerbation was 1.45% (44 patients), and only 14 patients died over 19 years. The major causes of severe acute exacerbation were HNC with liver metastases, severe liver cirrhosis, and sepsis-related multiple organ failure. No HNC patient experienced HBV or HCV reactivation induced severe acute exacerbation during CCRT. To our knowledge, there are no sufficient data about the prognosis of HBV or HCV infection in HNC patients. Zheng et al. reported that HBsAg-positive patients with NPC had similar survival outcomes as those with an HBsAg-negative status in the IMRT era [21]. Moreover, they also found that the HBsAg-positive NPC patients survived as long as the antiviral therapy group did [21]. Weng et al. also indicated that no statistically significant differences in the 5-year OS existed between the HbsAg-positive and HbsAg-negative groups [24]. Multivariate analysis revealed that the T stage, N stage, and clinical cancer stage were independent risk factors affecting the OS of the NPC patients [24]. These results are consistent with our study. Our study also showed that gender, primary tumor site (hypopharynx and others), T stage, N stage, and the AJCC tumor stage of cancer were independent prognostic factors of HNC patients to receive CCRT. Our research also found that the incidence rate of acute exacerbation and mortality of HNC patients with HBV or HCV was low. These results were also consistent with the findings of Shih et al. [25]. In Shih et al.’s research, no HbsAg-positive HNC patients experienced a severe acute exacerbation or mortality due to them not having antiviral prophylaxis. The reported HCV reactivation rate ranged from 6% to 10% in solid tumors, which is lower than that of hematologic malignancies during chemotherapy [19,26]. However, there were no precise data about the HCV reactivation rate in HNC patients with chronic HCV infection to receive chemotherapy. Our current study showed that 0.2% (6/3014) of the HNC patients with HCV infection had episodes of acute exacerbation during chemotherapy. This evidence supports our findings that antiviral prophylaxis did not affect the OS of the HNC patients with HBV or HCV carriers to receive CCRT due to the low incidence of HBV or HCV reactivation.

Our study found that biomarkers of liver cirrhosis (APRI and FIB-4) could predict the outcomes of the HNC patients receiving CCRT. APRI includes two routinely available biochemical and clinical parameters, aspartate aminotransferase (AST) and platelets (PLT), which evaluate the liver cirrhosis stage and liver function reserve [27]. The FIB-4 score consists of aspartate aminotransferase (ALT), PLT, age, and AST, which are used to assess the liver fibrosis status [28]. In oncology, APRI and FIB-4 can predict the prognosis of hepatocellular carcinoma [27,28]. Recently, FIB-4 was also shown to be a prognostic factor of gastric cancer [29]. As the literature review indicates, there are no studies about APRI or FIB-4 to predict the outcomes of HNC patients. Our study revealed that APRI and FIB-4 were significant independent prognostic factors of HNC patients receiving CCRT, regardless of their HBV or HCV infection status. A study by Chang et al. revealed that liver cirrhosis could predict the one-year survival rate of oral cancer patients [30]. The liver cirrhosis grade meaningfully influenced the therapeutic decisions and prognosis of people with primary liver cancer and non-liver malignancies. The patients with decompensated liver cirrhosis might be complicated, with portal hypertension, ascites, variceal bleeding, jaundice, hepatic encephalopathy, and a shorter life expectancy [31]. Our study provided valuable and accessible biomarkers of liver cirrhosis (APRI and FIB-4) to predict the survival of HNC patients receiving CCRT.

The Charlson Comorbidity Index (CCI) is calculated via the summation of weight scores for 19 medical conditions. Comorbidities are essential parameters for treatment decision-making and adjusting the outcome data via retrospective analyses. The CCI is one of the most common tools to assess the impacts of comorbidity on cancers and noncancer patients. In different types of cancer, a higher CCI has been associated with a poorer prognosis [15,32,33]. However, the correlation between the CCI and outcomes of HNC patients receiving CCRT has not previously been well evaluated. Our previous study revealed that a higher CCI predicted the poor overall survival and disease-free survival of HNC patients to receive radiotherapy or CCRT [34]. Our current study showed that the HNC patients with CCI ≥ 6 had a poorer survival rate than those with CCI< 6 while they received CCRT. A higher CCI indicates that the patients have multiple comorbidities and higher risks of non-cancer-related death.

The strength of this current study is the large sample size. The results suggest that antiviral prophylaxis provides no overall survival benefits to HNC patients with HBV or HCV infection while receiving CCRT. This first study indicates that APRI and FIB-4 are valuable biomarkers to predict the OS of HNC patients receiving CCRT. However, this study also has some limitations. This is a retrospective observational study conducted from 2001 to 2019. The cost of the antiviral prophylaxis of HBV in HNC patients before chemotherapy has been reimbursed since 2009, similar to anti-HCV agents in viremic patients since 2017, by the NHI providers in Taiwan. The amount of HNC patients who receive an antiviral treatment account for a small population of HNC patients who receive CCRT. In our hospital, the treatment modalities of most HNC patients were made at a multidisciplinary HNC cancer conference. Therefore, there could be a selection bias in the HNC patients receiving CCRT. The patients with severe comorbidities or liver cirrhosis should be excluded from receiving CCRT. Third, the HNC patients were identified mainly based on the International Classification of Diseases, Ninth and Tenth Revision codes. The HNC patients provided samples from oral, oropharynx, hypopharynx, larynx, and other sites, such as the nasopharynx. With these limitations, the tumor primary sites, comorbidity score (CCI), and liver cirrhosis data were adjusted via multivariate Cox regression to control the confounding factors. While comparing the antiviral effects of HNC patients to receive CCRT, the Cox regression analyses models with or without HBV or HCV all showed no survival difference due to antiviral prophylaxis. Moreover, gender, tumor stage, CCI, APRI, and FIB-4 were still associated with the OS of HNC patients during CCRT.

## 5. Conclusions

While the clinical guidelines, such as the American Society of Clinical Oncology guideline, recommend patients with solid tumors and hepatitis to initiate an antiviral treatment prior to chemotherapy in order to avert the acute exacerbation of chronic hepatitis, our study revealed that there were no survival differences for the HNC patients when treated with an antiviral therapy during CCRT, neither for an HBV or HCV infection. For these patients, factors such as gender, the initial tumor stage before CCRT, comorbidity index, and liver cirrhosis status emerged as significant prognostic factors for overall survival.

## Figures and Tables

**Table 1:** Demographic and clinical characteristics of the study. (*n* = 8224), AJCC: American Joint Committee on Cancer, CCI: Charlson Comorbidity Index; APRI: AST to Platelet Ratio Index.

Variables	Total Patient *n* = 8224	No Hepatitis B or C *n* = 5772	Hepatitis B or C without Antiviral Treatment in CCRT Course *n* = 1852	Hepatitis B or C with Antiviral Treatment in CCRT Course *n* = 600	*p*-Value
Age					
<52 y/o	4037	2808 (48.7%)	888 (48.0%)	341 (56.8%)	0.0004
≥52 y/o	4187	2964 (51.4%)	964 (52.1%)	259 (43.2%)	
Gender					
Male	7243	5021 (87.0%)	1683 (90.1%)	539 (89.8%)	<0.0001
Female	981	751 (13.0%)	169 (9.1%)	61 (10.2%)	
Primary site					
Oral	2599	1848 (30.0%)	661 (33.0%)	140 (23.3%)	<0.0001
Oropharynx	1321	881 (15.3%)	349 (18.8%)	91 (15.2%)	
Hypopharynx	1111	741 (12.8%)	274 (14.8%)	96 (16.0%)	
Larynx	498	342 (5.9%)	126 (6.8%)	30 (5%)	
Others	2695	1960 (34.0%)	492 (26.6%)	243 (40.5%)	
T stage					
1	1152	783 (13.6%)	270 (14.6%)	99 (16.5%)	0.0688
2	2129	1505 (26.1%)	468 (25.3%)	156 (26.0%)	
3	1428	977 (16.9%)	332 (17.9%)	119 (19.8%)	
4	3515	2507 (43.4%)	782 (42.2%)	226 (37.7%)	
N stage					
0	1748	1201 (20.8%)	436 (23.5%)	111 (18.5%)	0.0022
1	1899	1349 (23.4%)	403 (21.8%)	147 (24.5%)	
2	3712	2618 (45.4%)	838 (45.3%)	256 (42.7%)	
3	865	604 (10.5%)	175 (9.5%)	86 (14.3%)	
AJCC stage of cancer					
I and II	1415	981 (17.0%)	328 (17.7%)	106 (17.7%)	0.6332
III	1631	1140 (19.8%)	377 (20.4%)	114 (19.0%)	
IVA	3825	2686 (46.5%)	867 (46.8%)	272 (45.3%)	
IVB	1353	965 (16.7%)	280 (15.1%)	108 (18.0%)	
CCI					
0–5	8143	5743 (99.5%)	1808 (97.6%)	592 (98.7%)	<0.0001
≥6	81	29 (0.5%)	44 (2.38%)	8 (1.3%)	
APRI score					
<2	8124	5751 (99.6%)	1790 (96.7%)	583 (97.2%)	<0.0001
≥2	100	21 (0.4%)	62 (3.4%)	17 (2.8%)	
FIB-4 score					
<3.25	7869	5651 (97.9%)	1664 (89.9%)	554 (92.3%)	<0.0001
≥3.25	355	121 (2.1%)	188 (10.1%)	46 (7.7%)	

**Table 2:** Univariate and multivariate Cox proportional hazards of prognostic factors for HNC overall survival, Model A (*n* = 8224), including HNC patients without HBV or HCV infection.

Variables	Total Patient *n* = 8224	Hazard Ratio			
		Univariates	*p*-Value	Multivariates	*p*-Value
Age					
<52 y/o	4037	Ref.	<0.0001	Ref.	0.0982
≥52 y/o	4187	1.19 (1.11–1.28)		1.06 (0.99–1.15)	
Gender					
Male	7243	Ref.	<0.0001	Ref.	<0.0001
Female	981	0.46 (0.40–0.53)		0.64 (0.55–0.75)	
Primary site					
Oral	2599	Ref.		Ref.	
Oropharynx	1321	1.11 (1.00–1.23)	0.0491	1.11 (1.00–1.23)	0.0554
Hypopharynx	1111	1.32 (1.19–1.47)	<0.0001	1.25 (1.13–1.39)	<0.0001
Larynx	498	0.93 (0.80–1.09)	0.394	1.08 (0.92–1.26)	0.360
Others	2695	0.46 (0.41–0.51)	<0.0001	0.57 (0.52–0.64)	<0.0001
T stage					
1	1152	Ref.		Ref.	
2	2129	1.67 (1.43–1.94)	<0.0001	1.30 (1.12–1.52)	0.008
3	1428	1.98 (1.70–2.32)	<0.0001	1.66 (1.42–1.95)	<0.001
4	3515	2.77 (2.41–3.18)	<0.0001	2.06 (1.78–2.39)	<0.001
N stage					
0	1748	Ref.		Ref.	
1	1899	0.73 (0.64-.082)	<0.0001	1.00 (0.88–1.13)	0.9763
2	3712	1.28 (1.16–1.41)	<0.0001	1.31 (1.19–1.45)	<0.0001
3	865	1.47 (1.28–1.68)	<0.0001	2.09 (1.81–2.40)	<0.0001
AJCC stage of cancer					
I and II	1415	Ref.		Ref.	
III	1631	1.20 (1.03–1.39)	0.0177	1.22 (1.06–1.42)	0.0075
IVA	3825	2.19 (1.93–2.47)	<0.0001	1.73 (1.53–1.96)	<0.0001
IVB	1353	2.80 (2.44–3.22)	<0.0001	2.53 (2.20–2.90)	<0.0001
CCI					
0–5	8143	Ref.	<0.0001	Ref.	<0.0001
≥6	81	2.19 (1.62–2.97)		2.18 (1.61–2.95)	
APRI score					
<2	8124	Ref.	0.0002	Ref.	0.0044
≥2	100	1.68 (1.28–2.21)		1.49 (1.13–1.97)	
FIB-4 score					
<3.25	7869	Ref.	<0.0001	Ref.	<0.0001
≥3.25	355	2.04 (1.77–2.36)		1.82 (1.57–2.11)	
Viral hepatitis					
No viral hepatitis	5772	Ref.		Ref.	
Viral hepatitis without antiviral treatment in CCRT course	1852	1.02 (0.94–1.11)	0.638	0.90 (0.92–0.98)	0.0172
Viral hepatitis with antiviral treatment in CCRT course	600	0.90 (0.78–1.05)	0.196	0.90 (0.78–1.05)	0.194

**Table 3:** Univariate and multivariate Cox proportional hazards of prognostic factors for HNC overall survival, Model B (*n* = 2452), including HNC patients with HBV or HCV infection.

Variables	Total Patient *n* = 2452	Hazard Ratio			
		Univariates	*p*-Value	Multivariate	*p*-Value
Age					
<52 y/o	1129	Ref.	0.0947	Ref.	0.627
≥52 y/o	1153	1.12 (0.98–1.28)		1.03 (0.90–1.18)	
Gender					
Male	2222	Ref.	<0.0001	Ref.	0.0159
Female	230	0.44 (0.32–0.60)		0.67 (0.49–0.93)	
Primary site					
Oral	751	Ref.		Ref.	
Oropharynx	440	1.18 (0.98–1.41)	0.0832	1.18 (0.98–1.42)	0.0768
Hypopharynx	370	1.54 (1.28–1.86)	<0.0001	1.57 (1.30–1.89)	<0.0001
Larynx	156	1.05 (0.79–1.39)	0.7324	1.20 (0.91–1.60)	0.1995
Others	735	0.48 (0.39–0.58)	<0.0001	0.60 (0.48–0.74)	<0.0001
T stage					
1	369	Ref.		Ref.	
2	624	1.52 (1.17–1.98)	0.0016	1.12 (0.86–1.47)	0.4046
3	451	1.99 (1.53–2.60)	<0.0001	1.60 (1.22–2.09)	0.0007
4	1008	2.43 (1.91–3.09)	<0.0001	1.82 (1.41–2.33)	<0.001
N stage					
0	547	Ref.		Ref.	
1	550	0.72 (0.58–0.90)	0.0040	0.98 (0.78–1.23)	0.8698
2	1094	1.27 (1.07–1.50)	0.0072	1.31 (1.10–1.56)	0.0025
3	261	1.66 (1.30–2.11)	<0.0001	2.30 (1.80–2.95)	<0.0001
AJCC stage of cancer					
I and II	434	Ref.		Ref.	
III	491	1.27 (0.97–1.65)	0.0778	1.27 (0.98–1.66)	0.0729
IVA	1139	2.19 (1.75–2.72)	<0.0001	1.79 (1.43–2.24)	<0.0001
IVB	388	2.87 (2.44–3.68)	<0.0001	2.73 (2.12–3.51)	<0.0001
CCI					
0–5	2400	Ref.	0.0008	Ref.	0.0028
≥6	52	1.96 (1.32–2.89)		1.83 (1.23–2.71)	
APRI score					
<2	2373	Ref.	0.0001	Ref.	0.0013
≥2	79	1.83 (1.35–2.46)		1.65 (1.22–2.23)	
FIB-4 score					
<3.25	2218	Ref.	<0.0001	Ref.	<0.0001
≥3.25	234	2.12 (1.76–2.54)		1.84 (1.52–2.21)	
Viral hepatitis					
Viral hepatitis without antiviral treatment in CCRT course	1852	Ref.		Ref.	
Viral hepatitis with antiviral treatment < 84 days in CCRT course	97	0.91 (0.65–1.29)	0.598	1.07 (0.76–1.51)	0.6977
Viral hepatitis with antiviral treatment ≥ 84 days in CCRT course	503	0.88 (0.74–1.06)	0.1723	0.96 (0.80–1.15)	0.6804

## Data Availability

Restrictions apply to the availability of these data. Data were obtained from Chang Gung Research Database and are available with the permission of the Institutional Review Board (IRB) of the Kaohsiung and Chiayi branches of Chang Gung Memorial Hospital.
